# Genomic or Non-Genomic? A Question about the Pleiotropic Roles of Vitamin D in Inflammatory-Based Diseases

**DOI:** 10.3390/nu15030767

**Published:** 2023-02-02

**Authors:** Michael F. Holick, Luciana Mazzei, Sebastián García Menéndez, Virna Margarita Martín Giménez, Fatme Al Anouti, Walter Manucha

**Affiliations:** 1 Section on Endocrinology, Diabetes, Nutrition & Weight Management, Department of Medicine, School of Medicine, Boston University, Boston, MA 02118, USA; 2 Instituto de Bioquímica y Biotecnología, Facultad de Ciencias Médicas, Universidad Nacional de Cuyo, Mendoza 5500, Argentina; 3 Instituto de Medicina y Biología Experimental de Cuyo (IMBECU), Consejo Nacional de Investigaciones Científicas y Tecnológicas (CONICET), Mendoza 5500, Argentina; 4 Instituto de Investigaciones en Ciencias Químicas, Facultad de Ciencias Químicas y Tecnológicas, Universidad Católica de Cuyo, San Juan 5400, Argentina; 5 Department of Health Sciences, Zayed University, Abu Dhabi P.O. Box 144534, United Arab Emirates; 6 Área de Farmacología, Departamento de Patología, Facultad de Ciencias Médicas, Universidad Nacional de Cuyo, Mendoza 5500, Argentina

**Keywords:** vitamin D, genetic, genomic, epigenetic, immunity, inflammation

## Abstract

Vitamin D (vit D) is widely known for its role in calcium metabolism and its importance for the bone system. However, various studies have revealed a myriad of extra-skeletal functions, including cell differentiation and proliferation, antibacterial, antioxidant, immunomodulatory, and anti-inflammatory properties in various cells and tissues. Vit D mediates its function via regulation of gene expression by binding to its receptor (VDR) which is expressed in almost all cells within the body. This review summarizes the pleiotropic effects of vit D, emphasizing its anti-inflammatory effect on different organ systems. It also provides a comprehensive overview of the genetic and epigenetic effects of vit D and VDR on the expression of genes pertaining to immunity and anti-inflammation. We speculate that in the context of inflammation, vit D and its receptor VDR might fulfill their roles as gene regulators through not only direct gene regulation but also through epigenetic mechanisms.

## 1. Introduction

Vitamin D (vit D) is a fat-soluble vitamin that can be found in two different chemical structures: cholecalciferol (vit D3) or ergocalciferol (vit D2) [1]. The exposure to ultraviolet B rays, present in sunlight, is the most effective natural source of vit D. It also can be obtained by the consumption of some fatty fish (tuna, salmon, and mackerel), sun-exposed mushrooms, beef liver, cheese, egg yolks, fortified foods, or even by supplements [2].

The active form of the vit D (D represents either and/or vitamin D2 and vitamin D3) is 1,25-dihydroxyvitamin D (1,25(OH)2D) To become 1,25(OH)2D, first vit D is transported to the liver, where it is modified to 25-hydroxyvitamin D (25(OH)D) by CYP2R1, which is a 25-hydroxylase enzyme. Later, in the kidneys, 25(OH)D undergoes an additional hydroxylation by the enzyme 25 hydroxyvitamin D-1α-hydroxylase (CYP27B1), converting it to 1,25(OH)2D [3,4].

There is a lot of controversy about the optimal levels of 25(OH)D which is the measure of a person’s vitamin D status [5]. Most agreements indicate vit D deficiency is defined at values below 20 ng/mL, insufficiency between 21–29 ng/mL and sufficiency at values > 30 ng/mL, with the range between 40–60 ng/mL being preferred, and vit D intoxication at values above 150 ng/mL as recommended by the Endocrine Society Practice Guidelines on Vitamin D [6]. However, according to the Institute of Medicine (IOM), for the majority of the population, a minimum 25(OH)D serum level of 20 ng/mL (50 nmol/L) is considered adequate with limited sun exposure. Meanwhile, the risk of vit D deficiency is considered significant when the 25(OH)D serum concentrations are below 12 ng/mL (30 nmol/L) [7]. Such thresholds of serum 25(OH)D concentrations were established for maximum bone health and higher thresholds have been recommended for optimal overall health because of the strong association between vit D deficiency and cardiovascular diseases, certain types of cancer, type 2 diabetes mellitus, hypertension, metabolic syndrome, infectious diseases, autism, depression, autoimmune diseases, and others [8].

In this context, given that the inflammatory process is directly or indirectly involved in the main underlying mechanisms of most of the pathologies affecting current societies (such as those previously mentioned), the present review aims to summarize the pleiotropic effects of vit D, emphasizing its anti-inflammatory impact on different organ systems. Likewise, this review aims to provide a comprehensive overview of the genomic and non-genomic effects of vit D and vit D receptor (VDR) related to immunity and anti-inflammation. Deepening this knowledge would help develop new therapeutic alternatives for many diseases that constitute important causes of morbidity and mortality worldwide.

## 2. Pleiotropic Effects of Vitamin D in Inflammatory-Based Diseases

Numerous pleiotropic effects have been reported for vit D since its discovery a century ago. Today it is well known that in addition to its pivotal role in calcium homeostasis and bone metabolism, vit D has antibacterial, anti-proliferative, immunomodulatory, and anti-inflammatory actions, among other beneficial properties [9,10].

The nuclear VDR mediates two types of actions of 1,25(OH)2D:

-Non-genomic pathway: Ligand binding to the cytosolic population of VDRs triggers multiple intracellular signaling pathways or cascades, leading to immediate gene transcription-independent responses in cells. These pathways may also contribute to ultimately modulating the expression of target genes in the nucleus [2].

-Genomic pathway: The retinoic acid receptor (RXR) forms a heterodimer with the VDR bound to 1,25(OH)2D. The heterodimer translocates to the cell nucleus and binds to the vit D response element (VDRE) in the promoter regions of target genes, consequently regulating nuclear transcription. The binding of the ligand to VDR modifies the conformation of its LBD region, usually promoting the release of transcriptional co-repressors and the recruitment of co-activators and enzymes (histone acetylases) that modify the structure of chromatin, facilitating the transcriptional activation of genes. In other cases, the effect could be transcriptional repression; however, the exact mechanisms of such down-regulation are not well known [2].

VDR is expressed in the skin, parathyroid glands, adipocytes, small intestine, colon, and in other tissues [11,12]. Moreover, the VDRE is found in numerous genes, explaining the mechanisms associated with vit D, such as immune functions, intestinal barrier function, cell proliferation, gut microbiota modulation, and autophagy. Vit D immunomodulatory effects particularly have been widely investigated by several researchers [13,14,15]. Such effects were attributed to a direct relation to antigen-presenting cells and T-cells functions. Moreover, the lack of 1,25(OH)2D harms regulatory T-cells differentiation and weakens its functions, which may trigger autoimmune diseases [16,17].

In addition to its important anti-inflammatory role, vit D can exert protective effects against reactive oxygen species (ROS) and nitric oxide and may prevent oxidative damages [10]. It also reduces glutathione (GSH) synthesis, which is essential to glutathione peroxidase (GPX) activity, through the regulation of the gamma-glutamyl transpeptidase (g-GT) expression. Vit D cans also increase GSH formation by increasing glucose-6-phosphate dehydrogenase (G6PD), glutamate-cysteine ligase (CGL), and glutathione reductase activity [18].

Furthermore, vit D plays a role in antioxidants synthesis by regulating Klotho and Nrf2 (Nuclear factor erythroid 2–related factor 2) expression, both of which are essential to ROS signaling pathway function [19].

Vit D is involved in the regulation of muscle development and contractility through genomic actions, and stimulating the proliferation of muscle cells and their differentiation through the transcription of genes that express an increase in cellular DNA synthesis, followed by the induction of muscle proteins, specifically calcium and myosin binding proteins [20]. Vit D also exerts non-genomic actions by interacting with the specific muscle cell membrane receptor, which leads to the stimulation of adenyl cyclase and phospholipases C, D, and A2, and the activation of intracellular signaling pathways, such as MAPK (Mitogen-activated protein kinase) cascade, ultimately enhancing cell division [21]. In a recently published study by Max et al. [22], the authors observed that mice born from mothers with vit D deficiency had smaller muscle cells than mice whose mothers had adequate levels. It has long been observed that vit D deficiency leads to a myopathy characterized by proximal muscle weakness and atrophy, and the presence of VDR in skeletal muscle tissue has been supported by several studies which documented a decline in receptors with age [23].

### 2.1. The Role of Vitamin D in Inflammation at the Cardiovascular Level

Vit D deficiency is associated with an increase in serum levels of pro-inflammatory mediators, including IL-6 and tumor necrosis factor-alpha (TNF-α), which are related to both the development and progression of some vascular inflammatory pathologies [14,15,24,25]. In addition, a study carried out on obese patients revealed that reduced serum 25(OH)D concentrations were usually related to increased levels of other biomarkers of vascular inflammation such as high-sensitivity C reactive protein (hCRP) and fibrinogen [26]. Similar conclusions were informed for severely obese children [27,28]. The 25(OH)D levels below 20 ng/mL were associated with increased markers of oxidative/nitrosative stress, inflammation and endothelial activation, all of them indicators of cardiovascular risk. Moreover, in obese people, hyperleptidemia is usually observed. This disorder provokes vascular inflammation [29]. The 1,25(OH)2D3 pretreatment of cultured human umbilical vein-derived endothelial cells exposed to high concentrations of leptin with 1,25(OH)2D3 prevented the rise in the expression of vascular pro-inflammatory mediators caused by leptin, including CCL2, VCAM-1, and transforming growth factor β (TGF-β) [30]. Furthermore, Oma et al. [31] observed a higher presence of mononuclear cell infiltrates in the aortic adventitia of patients with coronary artery disease and vit D deficiency as compared with those with suiTable 25(OH)D levels. The adhesion of monocytes to endothelial cells represents an early stage in atherosclerosis development, thus, vit D supplementation would be a helpful tool in the prevention and treatment of atherosclerosis and other vascular inflammatory diseases [32]. Furthermore, it has also been suggested that vit D deficiency and the down-regulation of its receptor could be involved in aggravating vascular inflammation in pregnant women during preeclampsia (pregnancy hypertension). Interestingly, vit D supplementation reversed the inflammatory process in these patients [33]. These findings along with the significant number of VDR located in vascular smooth muscle cells (VSMCs) and endothelial cells indicates a crucial role of this endogenous compound in the regulation of inflammation, especially at the vascular level [34].

The location of VDR in VSMCs is of particular interest since these cells represent the majority of cell population in the blood vessel walls’ levels and they have a vital role in the advance of vascular inflammatory disease. New technologies have shown that VSMCs may switch their contractile phenotype to a macrophage-like phenotype, proving the plasticity of these cells. Thus, there is interplay among VSMCs, immune cells, and endothelial cells during the convoluted process of vascular inflammation where VSMCs can control, interact with, and influence the behavior of other cellular components of the blood vessel wall [35]. In this context, a study carried out using an in vitro model of endothelial inflammation (primary cultured human umbilical vein endothelial cells exposed to TNF-α) showed that the treatment with paricalcitol (a vit D analog) inhibited the increased expression of the intercellular adhesion molecule-1 (ICAM-1), vascular cell adhesion molecule-1 (VCAM-1), and fractalkine (a chemoattractant cytokine) in these cells [36]. Furthermore, in a clinical model of vascular inflammation (patients with an abdominal aneurysm), paricalcitol reduced the CD4+ T-helper and the T-cell (CD3+) content in aneurysm wall samples from these patients. Paricalcitol also prevented the rise in the levels of pro-inflammatory cytokines (IL-1β, IL-6, IL-8, and TNF-α) in cultured human aortic smooth muscle cells exposed to elevated concentrations of phosphate, indicating that active vit D derivatives have anti-inflammatory effects at the vascular level [37].

Likewise, VDRs also modulate the RAAS, inflammation, and fibrosis. Thus, their activation counteracts myocardial hypertrophy and hypertension [38]. In this regard, it is proposed that the activation of VDRs associated with Hsp70 (as a chaperone protein) could favor physiological cardiac remodeling after a myocardial infarction and reduce progression to heart failure [39].

In SHR rats, treatment with vit D analogs ameliorates left ventricular hypertrophy and improves left ventricular diastolic measures. Mizobuchi et al. [40] showed that combined therapy with enalapril and paricalcitol significantly decreased proteinuria, glomerulo-sclerotic index, and tubulointerstitial volume in uremic rats [41].

In addition, experimental studies suggest that active vit D analogues at low doses ameliorate myocardial renin overexpression and lower blood pressure, protect against aortic calcification and prevent cardiac/vascular remodeling [42].

Moreover, our previous work [38] in rats with a ureteral obstruction showed a reduction in myocardial VDR expression, and this fact, might be related to myocardial remodeling associated with an increase in arrhythmogenesis. Important to note, that paricalcitol protects against these changes by restoring myocardial VDR levels [38].

For its part, vit D deficiency at the cellular level produces a higher oxidative stress, inflammatory markers, and mitochondrial damage. Serum 25(OH)D concentrations below 25 ng/mL were related to an increase in vascular tone mediated by smooth muscle contraction, either through direct effects on vascular smooth muscle cells, up-regulation of the RAAS, and/or through modulation of calcium metabolism with secondary hyperparathyroidism; which predisposes patients to develop hypertrophy of the left ventricle and of the vascular wall, causing hypertension. The VDR plays a role at the mitochondrial level and the regulation of the respiratory chain, which would influence arterial remodeling, since its activation would reduce oxidative damage and preserve cell life. Such data implicates that maintaining adequate levels of vit D is important for protection against cardiovascular disease [42].

Several studies assessed the effect of calcitriol administration as an anti-inflammatory agent. On an apoE (−/−) mouse model of abdominal aneurysm induced by angiotensin-II (Ang-II) infusion, the oral treatment with calcitriol reduced the aneurysm formation and all altered parameters observed during abdominal aneurysm were decreased, including macrophages infiltration, expression of vascular endothelial growth factor (VEGF), angiogenesis, monocyte chemoattractant protein 1 (CCL2), CCL5, and CXCL1 chemokines and synthesis of matrix metalloproteinase-2 and 9 (MMP-2 and 9) [43]. In an in vitro model of human endothelial cells, the treatment caused inhibition of leukocyte-endothelial cell interactions induced by Ang-II, morphogenesis, and synthesis of endothelial pro-inflammatory and angiogenic chemokines mediated by VDRs [43,44].

In addition, vit D administration decreases the expression of pro-inflammatory and proatherogenic cytokines such as IL-2 and interferon-gamma (IFN-ɤ), which are responsible for the T-helper-1 cells activation and vascular inflammation [45]. Accordingly, clinical studies showed that vit D supplementation was able to reduce central blood pressure parameters in individuals with vit D deficiency, but not in individuals with adequate vit D status. It was also reported to improve arterial stiffness in overweight African Americans patients with vit D deficiency and to improve microvascular responses in African Americans, mitigating or preventing the development of cardiovascular dysfunction in this population [46].

### 2.2. Anti-Inflammatory Role of Vitamin D in the Gastrointestinal System

Vit D helps the intestine absorb calcium and phosphate and its deficiency is correlated with increased mucosal inflammation, leading to inflammatory bowel disease (IBD) [2]. However, administration of the vit D at a dose that raises and maintains a 25(OH)D of 30 ng/mL, may have the ability to reduce the disease. Moreover, VDR has been linked to the gut microbiota and its metabolites and its expression is down-regulated in Crohn’s disease (CD) and ulcerative colitis [2].

VDR is regulated by miRNAs, which are a class of small non-coding RNA (17–22 nucleotides) that regulates gene expression post-transcriptionally. It also inhibits transcription of ZO-1, claudin-5, and occludin genes and increases the tight junction protein claudin-2 which enhance intestinal permeability. Thus, lacking intestinal epithelial VDR regulation in inflamed intestine leads to hyperfunction of Claudin-2 and exaggerates the inflammatory responses in the intestines [47].

### 2.3. Vitamin D Role in Renal Protection

Several animal models have suggested a role for active vit D in albuminuria and kidney fibrosis. In fact, vit D analogues are able to affect blood pressure, proteinuria, and inflammation. A study carried out on VDR knockout mice reveals a rise in renin consequent to loss of normal suppression of the renin-angiotensin aldosterone system (RAAS) by vit D [48]. Thus, RAAS have been shown to play an important role in the progression of chronic kidney disease (CKD) and low levels of 25(OH)D and 1,25 (OH)2D were correlated to be predictors of disease progression and death in patients with CKD and End Stage Renal Disease [49].

In this regard, in previous investigations, the expression of VDR and genes associated with nephrogenesis in spontaneous hypertension rats (SHR) from week 0 to 8 of life were analyzed. Hypertension in these rats is known to develop at about 6 weeks of age [50]. We observed a decrease in the expression of the nephrogenic gene, wt-1, and VDR by week 4, before the establishment of arterial hypertension, suggesting that the alteration in the kidney occurs previous to the increase in blood pressure [51].

Moreover, our group has found in adult SHR rats that the induction of VDR modulates an increase in Hsp70 levels, with a decrease in the angiotensin II receptor, type 1 (AT1) expression, providing renal protection [41]. Of interest, Hsp70 effects on the VDR may also accentuate repressive anti-inflammatory signaling [52].

### 2.4. The Role of Vitamin D in the Nervous System

As early as the early 1980s, attempts were made to find a relationship between the nervous system and vit D, starting with the question of whether vit D was able to cross the blood-brain barrier [53]. Today the questions revolve around the relationship of vit D status and its interaction with antioxidant mechanisms, complex immunomodulatory systems, and neurotrophic factors among others [19,54]. As a result, vit D has a key part in the process of neuro-inflammation, cognitive decline, and neurodegeneration [55]. In line with this, it has recently been reported that vit D has a neuroprotective function in aging cognitive decline [56]. This is one of the reasons why many human dietary supplements include vit D alone or in combination with other compounds that have antioxidant effects. A closer look into the different cell types of the nervous system, VDR are found in neurons, astrocytes, and microglia in the central nervous system [54]. Furthermore, using the 25-hydroxyvitamin D-1alpha-hydroxylase (CYP27B1) and 25-hydroxyvitamin D-24-hydroxylase (CYP24A1) enzymes, some of these cells may synthesize and catabolize 1,25(OH)2D. The unequal distribution of 1,25(OH)2D and 25(OH) D in different parts of the brain shows that vit D and its metabolism in the CNS might either function in a paracrine or autocrine manner [57].

Many studies have discovered that appropriate amounts of vit D reduce oxidative stress and brain inflammation, resulting in diverse neuroprotective benefits [58]. The VDR is also implicated in the neuroprotective effects of neurosteroids. Furthermore, calcitriol has been shown to stimulate VDR expression, down-regulate NOX2, and inhibit cellular death rate [59], suggesting that VDR-activated ERK1/2 activation may contribute to neuronal apoptosis prevention [60]. On the other hand, findings show that vit D shortage causes significant changes in microglia, implying that these cells may play a role in the sensory dysfunctions associated with hypovitaminosis D [61].

The human brain produces 1,25(OH)2D3, which affects a variety of brain areas, including the prefrontal cortex, hippocampus, cingulate gyrus, thalamus, hypothalamus, and substantia nigra. The 1,25(OH)2D3 lowers oxidative stress, inhibits inflammation, offers neuroprotection, down-regulates inflammatory mediators, and up-regulates neurotrophins in neurons [62].

The Klotho gene, which was initially discovered as an ‘aging suppressor’ in mice, encodes the antiaging protein Klotho. Klotho deficiency is linked to early mortality and rapid aging, but its overexpression is linked to longevity. Klotho protein is involved in the control of a number of biological processes, including calcium-phosphate balance, PTH, and vit D metabolism [63]. The precise chemical pathways through which 1,25(OH)2D3 and Klotho protein exerts its activities in the brain are yet unknown; however, the relationship between them might be both genomic and non-genomic, but the interplay processes are mainly unknown [63].

For all the above reasons, we could hypothesize that the therapeutic use of 1,25(OH)2D3 or similar agonists may have considerable promise due to its documented function in neuroinflammation, neurodegenerative diseases, and neuropsychiatric disorders.

Depression is a frequent mental illness in the elderly that lowers quality of life and increases morbidity and death. Vit D may play a role in the onset and treatment of depression as a neuro-steroid hormone [64]. One of the proposed mechanisms by which this neurohormone exerts its action is its relationship with serotonin and dopamine levels in the brain [65].

### 2.5. The Role of Vitamin D in Autoimmunity

Clinical studies have suggested that VDR polymorphisms and vit D deficiency is related to the development and progression of several autoimmune diseases, such as rheumatoid arthritis, systemic lupus erythematosus, multiple sclerosis, and autoimmune endocrine disorders (e.g., Hashimoto thyroiditis, type-1 diabetes mellitus (T1DM), Addison’s disease, and Graves’ disease) [66]. As mentioned, due to its great ability to bind to VDR and act as a transcriptional factor, vit D may modulate gene expression and, consequently, exert immunomodulatory effects on immune cells. Vit D has the capability to inhibit the production of Th17 cytokine, improve Treg activity, stimulate NKT cell functions, inhibit Th1, and induce the production of Th2 cytokine, and thereby shift T cells toward Th2 profile [67]. However, the role of vit D supplementation in the improvement of autoimmune diseases remains unclear. Therefore, additional studies are needed to know the potential underlying mechanisms involved [68]. Of note, all the clinical studies performed so far, only demonstrate correlations. Thus, it is difficult to establish whether the low 25(OH)D3 level is the cause or the consequence of autoimmune diseases [69]. Despite several studies having demonstrated a beneficial effect of vit D supplementation in autoimmune diseases, there are also some studies that did not show any effect on the main parameters of this kind of diseases. This could be due to differences in the supplementation strategy or the individual characteristics of the subjects included in the study, which are aspects that should be addressed in a properly way at the moment of designing multicenter clinical trials [70,71].

## 3. Genetic and Epigenetic Regulation of Inflammation by Vitamin D

Epigenetics might explain the interplay between environment and genetics in the development, progression, pathogenicity of disease, and the response to treatments. Epigenetics changes occur in the following ways: DNA methylation, which generates methyl cytosine in CpG dinucleotides sites leading to transcriptional silencing, histone modifications, chromatin remodeling, and noncoding RNAs regulation [29]. RNAs are untranslated transcripts that play an important role in the post-transcriptional regulation of gene expression and can be classified into short, mid, and long based on their length. Among the noncoding RNAs, long noncoding RNAs (lncRNAs) regulate gene expression by interacting with DNA, messenger RNAs (mRNAs), and proteins, while microRNAs (miRNAs) mediate the post-transcriptional repression or mRNA degradation in an epigenetic mechanism [72].

One of the most usual processes observed during vascular inflammation is histone acetylation/deacetylation. Acetylation of histones (by histone acetyltransferases) is the addition of positively charged acetyl groups to amino acid residues, which neutralizes the negative charges of DNA reducing the affinity of histones to DNA, and leading to a more relaxed chromatin state to allow higher accessibility to the transcriptional machinery [73]. Likewise, the opposite mechanism (deacetylation of histones) is carried out by histone deacetylases (HDACs) [74]. Of interest, alterations in any of these systems may produce abnormal activation or silencing of specific genes, which favors vascular disease onset. In addition, transgenerational transmission of epigenetic signaling is informed to precede vascular damage in children and young adults [75]. Thus, it is needed to investigate the existing link between epigenetics and inflammation in the vasculature to understand how epigenetic changes may influence these biological processes and how they could be managed to inhibit the inflammatory cascade at the vascular level [76].

Notably, during pregnancy, most calcitriol is produced in the maternal kidneys and a small proportion in the placenta [77]. Vit D induces epigenetic changes that modify the way the placenta responds to this compound and its metabolites and can even reduce the epigenetic changes associated with gestational aging and also plays a role in modifying the immune response of the fetus [78]. Moreover, this research suggests that maternal 25(OH)D levels influence gene expression profiles, and these changes could contribute to fetal immune imprinting and reducing allergic sensitization in the first years of life [78].

It is relevant to highlight that pregnant women are at an increased risk of developing vit D deficiency, and this is associated with adverse health consequences among their offspring, such as hypertension, hypocalcemia, deficient postnatal growth, and autoimmune diseases, among others [79]. Regarding this, exposure of male and female Sprague-Dawley rats to a vit D-free diet before mating led to an increase in systolic and diastolic blood pressure in their offspring, in addition to the hypermethylation of the Pannexin-1 (Panx1), a gene responsible for endothelial relaxation of large blood vessels [80]. Using the same model, Zhang and coworkers also showed that maternal vitamin D-free diet (and ultraviolet-free light) during pregnancy may cause insulin resistance in the offspring, which is accompanied by persistent increased inflammation. The vit D-free diet caused a significant increase in NFKBIA methylation at the CpG site +331 in the offspring. NFKBIA protein interacts with REL dimers to block NF-kappa-B/REL complexes which are involved in inflammatory pathways. Hence, through epigenetic modifications, the persistent lower Iκbα expression levels in the progeny stimulates the activation of NF-κB signaling which, in turn, leads to inflammation with possible implication for the insulin resistance in adulthood [81]. These studies emphasize the relevance of vit D supplementation for the prevention of epigenetically induced vascular inflammatory pathologies.

In fact, there is strong evidence that vit D and epigenetic mechanism programming is highly related. As previously mentioned, calcitriol acts through its binding to the nuclear-cytosolic VDR. After the heterodimer VDR-RXR is formed, genomic action is performed via docking of the VDR-RXR complex to the VDRE in the proximal region of promoter genes modulated by vitamin D [82,83]. The level of chromatin accessibility in the VDRE regions is crucial for VDR target gene transcription. Hence, VDREs cannot be accessed by the active VDR-RXR complex if it is inside a hypermethylated heterochromatin region. Therefore, the transcriptional control of VDR-responsive genes is dependent on three mechanisms: vit D availability, VDREs accessibility, and VDR expression levels. All these actions are regulated by environmental, genetic, and epigenetic factors. From the environmental factors, sun exposure, pollution, diet, and infection are able to regulate the VDR mainly by altering the levels of vit D [84,85].

VDR expression is differently correlated with inflammatory cytokines expression and miRNAs regulation, mainly miRNA-21, -214, and -125. A single nucleotide polymorphism of the VDR gene constitutes a risk factor for coronary artery disease [86]. In macrophages, which are essential immune cells during the vascular inflammatory response, upregulation of miRNA-155 is associated with LPS induced hyper-inflammatory process by the inhibition of suppressor of cytokine signaling 1 (SOCS1). On the contrary, 1,25-dihydroxycholecalciferol-VDR signaling has been demonstrated to downregulate BIC gene transcription by blocking the activation of NF-κB, leading to decreased miRNA155 levels and increased SOCS1 translation [87]. In addition, upregulation of epithelial VCAM-1, ICAM-1, and IL-6 is related to inflammation and atherogenesis in an in vivo mouse model of VDR deletion [88]. VDR is directly involved in the suppression of NF-κB activation, which may explain, at least in part, the VDR-mediated anti-inflammatory effects of vit D in cardiovascular pathologies [88,89]. Most of the studies on vit D and vascular inflammatory process focus on immune cells such as monocytes/macrophages or T cells activated by LPS. In this context, Gynther and coworkers demonstrated that LPS-treated human monocytes exposed to vit D (10 nM, cholecalciferol) downregulated the pro-inflammatory IL-12B gene by VDR-RXR binding. By the recruitment of co-repressor NCOR2/SMRT and HDAC3, a significant reduction in histone 4 acetylation and an increase in histone 3 trimethylation were observed at the IL-12B promoter [90]. Additionally, VDR-RXR binding to the nuclear factor needed for the activation of T cell (NFAT) sites caused the dissociation of acetylated histone H4 from IL17A promoter and recruitment of HDAC2 to the NFAT sites, thus supporting the role of 1,25(OH)2D in attenuating the pathogenesis of vascular inflammation [91]. By binding to the TLR4, LPS triggers mitogen-activated protein kinases (MAPK) and NF-κB signaling activation, causing the expression of TNF, IL1B, IL6, and IL8 genes [21]. Curiously, when monocytes obtained from patients with type 1 diabetes mellitus with microvascular complications were pre-incubated with vit D (0.1 μmol/L), a reduction in their LPS-activated TLR4 expression and cytokine levels was observed [92]. Moreover, Zhang et al. [93] found that vit D dose dependently (15–70 ng/mL) inhibits LPS-induced cytokine synthesis by the upregulation of MAPK phosphatase 1 (MKP-1) in human monocytes and murine bone marrow-derived macrophages. Notably, increased VDR binding to a putative VDRE in the MKP-1 promoter and histone H4 acetylation close the VDRE site led to MAPK inactivation, thereby reducing p38 activation and cytokine production. Another study demonstrated that 1,25(OH)2D has no distinguishable effect on p38 phosphorylation in macrophages, suggesting that the downregulation of the pro-inflammatory COX-2 happens in a MAPK-independent manner. In this study, vit D blocked Akt/NF-κB/COX-2 axis-mediated proinflammatory cytokines by binding VDR to a functional VDRE in the thioesterase superfamily member 4 (THEM4) promoters, thereby helping to the cardiovascular protective effects of vit D [94]. Epidemiological studies informed that vit D deficiency (25(OH)D < 15 ng/ mL) could be considered a sign of cardiovascular risk, mostly in people with hypertension [95]. In fact, there is an association between vit D deficiency and inflammatory disorders in humans; this is so usual it is considered a global issue [96]. Recently, Wimalawansa [97] recapitulated the role of vit D on oxidative stress, epigenetics, gene expression, inflammation, and the aging process. These aging-related processes seem to happen at lower rates in individuals with normal vit D status. Severe vit D deficiency (25(OH)D ≤ 10 ng/mL) seems to be associated with methylation in leukocyte DNA, mainly in MAPRE2 and DIO3 genes, both correlated with tumor development [98]. All these studies support the genetic and epigenetic modulation of vit D and its relevance in inflammation.

## 4. Conclusions

It is common to associate vit D with skeletal homeostasis. However, new roles are constantly emerging for vit D with closer investigations. VDR is linking at hundreds of sites in the genome and is associated with the regulation of more than 60 genes. Moreover, vit D (and its active derivatives) not only play a role in the genetic regulation of many genes, but also in the epigenetic regulation. In fact, the epigenetic machinery can be altered by vit D, acting through multiple genetic mechanisms. Gene expression can be modified by numerous miRNAs working as epigenetic modulators of VDR, also by methylation and histone acetylation/deacetylation [2].

Vit D is involved in processes related to the development and progression of some inflammatory diseases, and has the ability to alter serum levels of both proinflammatory and anti-inflammatory mediators.

Beyond the known intimate relationship between vit D and the gastrointestinal tract, we can highlight a bidirectional communication between the VDR and the intestinal microbiota. Both participate in an autoregulation in which different types of post-transcriptional mechanisms and their relationship with intestinal permeability are also involved.

Moreover, vit D has protective effects in CKD and hypertension, and one of the mechanisms by which it carries out this function is through the alteration of the RAAS system.

On the other hand, with new research findings, more insight is being obtained about the relationship between vit D and different pathologies of the nervous system. Most of these are focused on vit D and its relationship with oxidative stress. We know that oxidative stress is one of the best-known starting points of different neuroinflammatory, neurodegenerative, and neuropsychiatric processes. It is clear that vit D has implications in general health and this fact invites researchers to continue looking for functions and delving into the study of other physiological actions and how its deficiency is associated with numerous pathologies beyond those explained here, such as diabetes, obesity, and cancer. Thus, we speculate that vit D, now considered as calciotropic hormone, may later be considered a hormone with multisystemic action.

## Data Availability

No application.

## References

[1] Pop T.L., Sîrbe C., Benţa G., Mititelu A., Grama A. (2022). The role of vitamin D and vitamin D binding protein in chronic liver Diseases. Int. J. Mol. Sci.

[2] Battistini C., Ballan R., Herkenhoff M.E., Saad S.M.I., Sun J. (2020). Vitamin D modulates intestinal microbiota in inflammatory bowel diseases. Int. J. Mol. Sci..

[3] Holick M.F. (2007). Vitamin D deficiency. N. Engl. J. Med..

[4] Slominski A.T., Chaiprasongsuk A., Janjetovic Z., Kim T.K., Stefan J., Slominski R.M., Hanumanthu V.S., Raman C., Qayyum S., Song Y. (2020). Photoprotective properties of vitamin D and lumisterol hydroxyderivatives. Cell Biochem. Biophys..

[5] Giustina A., Bouillon R., Binkley N., Sempos C., Adler R.A., Bollerslev J., Dawson-Hughes B., Ebeling P.R., Feldman D., Heijboer A. (2020). Controversies in vitamin D: A statement from the third international conference. JBMR Plus.

[6] Holick M.F., Binkley N.C., Bischoff-Ferrari H.A., Gordon C.M., Hanley D.A., Heaney R.P., Murad M.H., Weaver C.M. (2011). Endocrine society. Evaluation, treatment, and prevention of vitamin D deficiency: An endocrine society clinical practice guideline. J. Clin. Endocrinol. Metab..

[7] Institute of Medicine (2011). Dietary Reference Intakes for Calcium and Vitamin D.

[8] Bouillon R., Manousaki D., Rosen C., Trajanoska K., Rivadeneira F., Richards J.B. (2022). The health effects of vitamin D supplementation: Evidence from human studies. Nat. Rev. Endocrinol..

[9] Charoenngam N., Holick M.F. (2020). Immunologic effects of vitamin D on human health and disease. Nutrients.

[10] Vojinovic J. (2014). Vitamin D receptor agonists’ anti-inflammatory properties. Ann. N. Y. Acad. Sci..

[11] Sirajudeen S., Shah I., Al Menhali A. (2019). A narrative role of vitamin D and its receptor: With current evidence on the gastric tissues. Int. J. Mol. Sci..

[12] Charoenngam N., Shirvani A., Kalajian T.A., Song A., Holick M.F. (2020). The effect of various doses of oral vitamin D_3_ supplementation on gut microbiota in healthy adults: A randomized, double-blinded, dose-response study. Anticancer Res..

[13] Ferder L., Martín Giménez V.M., Inserra F., Tajer C., Antonietti L., Mariani J., Manucha W. (2020). Vitamin D supplementation as a rational pharmacological approach in the COVID-19 pandemic. Am. J. Physiol. Lung Cell. Mol. Physiol..

[14] Martín Giménez V.M., Inserra F., Ferder L., García J., Manucha W. (2021). Vitamin D deficiency in African Americans is associated with a high risk of severe disease and mortality by SARS-CoV-2. J. Hum. Hypertens..

[15] Martín Giménez V.M., Lahore H., Ferder L., Holick M.F., Manucha W. (2021). The little-explored therapeutic potential of nanoformulations of 1,25-dihydroxyvitamin D_3_ and its active analogs in prevalent inflammatory and oxidative disorders. Nanomedicine.

[16] Ismailova A., White J.H. (2022). Vitamin D, infections and immunity. Rev. Endocr. Metab. Disord..

[17] White J.H. (2022). Emerging roles of vitamin D-induced antimicrobial peptides in antiviral innate immunity. Nutrients.

[18] Jain S.K., Micinski D. (2013). Vitamin D upregulates glutamate cysteine ligase and glutathione reductase, and GSH formation, and decreases ROS and MCP-1 and IL-8 secretion in high-glucose exposed U937 monocytes. Biochem. Biophys. Res. Commun..

[19] Câmara A.B., Brandão I.A. (2021). The relationship between vitamin D deficiency and oxidative stress can be independent of age and gender. Int. J. Vitam. Nutr. Res..

[20] Dzik K.P., Kaczor J.J. (2019). Mechanisms of vitamin D on skeletal muscle function: Oxidative stress, energy metabolism and anabolic state. Eur. J. Appl. Physiol..

[21] Hii C.S., Ferrante A. (2016). The non-genomic actions of vitamin D. Nutrients.

[22] Max D., Brandsch C., Schumann S., Kühne H., Frommhagen M., Schutkowski A., Hirche F., Staege M.S., Stangl G.I. (2014). Maternal vitamin D deficiency causes smaller muscle fibers and altered transcript levels of genes involved in protein degradation, myogenesis, and cytoskeleton organization in the newborn rat. Mol. Nutr. Food Res..

[23] Gómez de Tejada Romero M.J. (2014). Acciones extraóseas de la vitamina D. Rev. Osteoporos. Metab. Miner..

[24] de Las Heras N., Martín Giménez V.M., Ferder L., Manucha W., Lahera V. (2020). Implications of oxidative stress and potential role of mitochondrial dysfunction in COVID-19: Therapeutic effects of vitamin D. Antioxidants.

[25] Martín Giménez V.M., Ferder L., Inserra F., García J., Manucha W. (2020). Differences in RAAS/vitamin D linked to genetics and socioeconomic factors could explain the higher mortality rate in African Americans with COVID-19. Ther. Adv. Cardiovasc. Dis..

[26] Bellia A., Garcovich C., D’Adamo M., Lombardo M., Tesauro M., Donadel G., Gentileschi P., Lauro D., Federici M., Lauro R. (2013). Serum 25-hydroxyvitamin D levels are inversely associated with systemic inflammation in severe obese subjects. Intern. Emerg. Med..

[27] Zakharova I., Klimov L., Kuryaninova V., Nikitina I., Malyavskaya S., Dolbnya S., Kasyanova A., Atanesyan R., Stoyan M., Todieva A. (2019). Vitamin D insufficiency in overweight and obese children and adolescents. Front. Endocrinol..

[28] Reyman M., Verrijn Stuart A.A., van Summeren M., Rakhshandehroo M., Nuboer R., De Boer F.K., Van Den Ham H.J., Kalkhoven E., Prakken B., Schipper H. (2014). Vitamin D deficiency in childhood obesity is associated with high levels of circulating inflammatory mediators, and low insulin sensitivity. Int. J. Obes..

[29] Martín Giménez V.M., Chuffa L.G.A., Simão V.A., Reiter R.J., Manucha W. (2022). Protective actions of vitamin D, anandamide and melatonin during vascular inflammation: Epigenetic mechanisms involved. Life Sci..

[30] Teixeira T.M., da Costa D.C., Resende A.C., Soulage C.O., Bezerra F.F., Daleprane J.B. (2017). Activation of Nrf2-antioxidant signaling by 1,25-dihydroxycholecalciferol prevents leptin-induced oxidative stress and inflammation in human endothelial cells. J. Nutr..

[31] Oma I., Andersen J.K., Lyberg T., Molberg Ø., Whist J., Fagerland M., Almdahl S., Hollan I. (2017). Plasma vitamin D levels and inflammation in the aortic wall of patients with coronary artery disease with and without inflammatory rheumatic disease. Scand. J. Rheumatol..

[32] Tay H.M., Yeap W.H., Dalan R., Wong S.C., Hou H.W. (2020). Increased monocyte-platelet aggregates and monocyte-endothelial adhesion in healthy individuals with vitamin D deficiency. FASEB J..

[33] Poniedziałek-Czajkowska E., Mierzyński R. (2021). Could vitamin D be effective in prevention of preeclampsia?. Nutrients.

[34] Gouni-Berthold I., Berthold H.K. (2021). Vitamin D and vascular disease. Curr. Vasc. Pharmacol..

[35] Sorokin V., Vickneson K., Kofidis T., Woo C.C., Lin X.Y., Foo R., Shanahan C.M. (2020). Role of vascular smooth muscle cell plasticity and interactions in vessel wall inflammation. Front. Immunol..

[36] Lee A.S., Jung Y.J., Thanh T.N., Lee S., Kim W., Kang K.P., Park S.K. (2016). Paricalcitol attenuates lipopolysaccharide-induced myocardial inflammation by regulating the NF-κB signaling pathway. Int. J. Mol. Med..

[37] Martínez-Moreno J.M., Herencia C., de Oca A.M., Díaz-Tocados J.M., Vergara N., Gómez-Luna M.J., López-Argüello S.D., Camargo A., Peralbo-Santaella E., Rodríguez-Ortiz M.E. (2017). High phosphate induces a pro-inflammatory response by vascular smooth muscle cells and modulation by vitamin D derivatives. Clin. Sci..

[38] Diez E.R., Altamirano L.B., García I.M., Mazzei L., Prado N.J., Fornes M.W., Carrión F.D.C., Zumino A.Z.P., Ferder L., Manucha W. (2015). Heart remodeling and ischemia-reperfusion arrhythmias linked to myocardial vitamin d receptors deficiency in obstructive nephropathy are reversed by paricalcitol. J. Cardiovasc. Pharmacol. Ther..

[39] Sanz R.L., Mazzei L., Manucha W. (2019). Implications of the transcription factor WT1 linked to the pathologic cardiac remodeling post-myocardial infarction. Clin. Investig. Arterioscler..

[40] Mizobuchi M., Morrissey J., Finch J.L., Martin D.R., Liapis H., Akizawa T., Slatopolsky E. (2007). Combination therapy with an angiotensin-converting enzyme inhibitor and a vitamin D analog suppresses the progression of renal insufficiency in uremic rats. J. Am. Soc. Nephrol. JASN.

[41] García I.M., Altamirano L., Mazzei L., Fornés M., Cuello-Carrión F.D., Ferder L., Manucha W. (2014). Vitamin D receptor-modulated Hsp70/AT1 expression may protect the kidneys of SHRs at the structural and functional levels. Cell Stress Chaperones.

[42] Sanz R., Mazzei L., Santino N., Ingrasia M., Manucha W. (2020). Vitamin D-mitochondria cross-talk could modulate the signaling pathway involved in hypertension development: A translational integrative overview. Clin. Investig. Arterioscler..

[43] Martorell S., Hueso L., Gonzalez-Navarro H., Collado A., Sanz M.J., Piqueras L. (2016). Vitamin D receptor activation reduces angiotensin-ii-induced dissecting abdominal aortic aneurysm in apolipoprotein E-knockout mice. Arterioscler. Thromb. Vasc. Biol..

[44] Wimalawansa S.J. (2018). Vitamin D and cardiovascular diseases: Causality. J. Steroid Biochem. Mol. Biol..

[45] Bennett A.L., Lavie C.J. (2017). Vitamin D metabolism and the implications for atherosclerosis. Adv. Exp. Med. Biol..

[46] Raed A., Bhagatwala J., Zhu H., Pollock N.K., Parikh S.J., Huang Y., Havens R., Kotak I., Guo D.-H., Dong Y. (2017). Dose responses of vitamin D3 supplementation on arterial stiffness in overweight African Americans with vitamin D deficiency: A placebo controlled randomized trial. PLoS ONE.

[47] Sun J., Zhang Y.G. (2022). Vitamin D receptor influences intestinal barriers in health and disease. Cells.

[48] Xiang W., Kong J., Chen S., Cao L.-P., Qiao G., Zheng W., Liu W., Li X., Gardner D.G., Li Y.C. (2005). Cardiac hypertrophy in vitamin D receptor knockout mice: Role of the systemic and cardiac renin-angiotensin systems. Am. J. Physiol. Endocrinol. Metab..

[49] Chau Y.Y., Kumar J. (2012). Vitamin D in chronic kidney disease. Indian J. Pediatr..

[50] Dornas W.C., Silva M.E. (2011). Animal models for the study of arterial hypertension. J. Biosci..

[51] Mazzei L., García M., Calvo J.P., Casarotto M., Fornés M., Abud M.A., Cuello-Carrión D., Ferder L., Manucha W. (2016). Changes in renal WT-1 expression preceding hypertension development. BMC Nephrol..

[52] Mazzei L., Docherty N.G., Manucha W. (2015). Mediators and mechanisms of heat shock protein 70 based cytoprotection in obstructive nephropathy. Cell Stress Chaperones.

[53] Gascon-Barré M., Huet P.M. (1983). Apparent [3H]1,25-dihydroxyvitamin D3 uptake by canine and rodent brain. Am. J. Physiol..

[54] Lee P.W., Selhorst A., Lampe S.G., Liu Y., Yang Y., Lovett-Racke A.E. (2020). Neuron-specific vitamin d signaling attenuates microglia activation and CNS autoimmunity. Front. Neurol..

[55] Menéndez S.G., Martín Giménez V.M., Holick M.F., Barrantes F.J., Manucha W. (2022). COVID-19 and neurological sequelae: Vitamin D as a possible neuroprotective and/or neuroreparative agent. Life Sci..

[56] Bayat M., Kohlmeier K.A., Haghani M., Haghighi A.B., Khalili A., Bayat G., Hooshmandi E., Shabani M. (2021). Co-treatment of vitamin D supplementation with enriched environment improves synaptic plasticity and spatial learning and memory in aged rats. Psychopharmacology.

[57] Gáll Z., Székely O. (2021). Role of vitamin D in cognitive dysfunction: New molecular concepts and discrepancies between animal and human findings. Nutrients.

[58] Kim H.A., Perrelli A., Ragni A., Retta F., De Silva T.M., Sobey C.G., Retta S.F. (2020). Vitamin D deficiency and the risk of cerebrovascular disease. Antioxidants.

[59] Cui C., Xu P., Li G., Qiao Y., Han W., Geng C., Liao D., Yang M., Chen D., Jiang P. (2019). Vitamin D receptor activation regulates microglia polarization and oxidative stress in spontaneously hypertensive rats and angiotensin II-exposed microglial cells: Role of renin-angiotensin system. Redox Biol..

[60] Guo X., Yuan J., Wang J., Cui C., Jiang P. (2019). Calcitriol alleviates global cerebral ischemia-induced cognitive impairment by reducing apoptosis regulated by VDR/ERK signaling pathway in rat hippocampus. Brain Res..

[61] Alessio N., Belardo C., Trotta M.C., Paino S., Boccella S., Gargano F., Pieretti G., Ricciardi F., Marabese I., Luongo L. (2021). Vitamin D deficiency induces chronic pain and microglial phenotypic changes in mice. Int. J. Mol. Sci..

[62] Lang F., Ma K., Leibrock C.B. (2019). 1,25(OH)2D3 in brain function and neuropsychiatric disease. Neurosignals.

[63] Dërmaku-Sopjani M., Kurti F., Xuan N.T., Sopjani M. (2021). Klotho-dependent role of 1,25(OH)2D3 in the brain. Neurosignals.

[64] Zech L.D., Scherf-Clavel M., Daniels C., Schwab M., Deckert J., Unterecker S., Herr A.S. (2021). Patients with higher vitamin D levels show stronger improvement of self-reported depressive symptoms in psychogeriatric day-care setting. J. Neural. Transm..

[65] Seyedi M., Gholami F., Samadi M., Djalali M., Effatpanah M., Yekaninejad M.S., Hashemi R., Abdolahi M., Chamari M., Honarvar N.M. (2019). The effect of vitamin D3 supplementation on serum BDNF, dopamine, and serotonin in children with attention-deficit/hyperactivity disorder. CNS Neurol. Disord. Drug Targets.

[66] Fletcher J., Bishop E.L., Harrison S.R., Swift A., Cooper S.C., Dimeloe S.K., Raza K., Hewison M. (2022). Autoimmune disease and interconnections with vitamin D. Endocr. Connect..

[67] Yang C.Y., Leung P.S., Adamopoulos I.E., Gershwin M.E. (2013). The implication of vitamin D and autoimmunity: A comprehensive review. Clin. Rev. Allergy Immunol..

[68] Ao T., Kikuta J., Ishii M. (2021). The effects of vitamin D on immune system and inflammatory diseases. Biomolecules.

[69] Bellan M., Andreoli L., Mele C., Sainaghi P.P., Rigamonti C., Piantoni S., De Benedittis C., Aimaretti G., Pirisi M., Marzullo P. (2020). Pathophysiological role and therapeutic implications of vitamin D in autoimmunity: Focus on chronic autoimmune diseases. Nutrients.

[70] Dankers W., Colin E.M., van Hamburg J.P., Lubberts E. (2017). Vitamin D in autoimmunity: Molecular mechanisms and therapeutic potential. Front. Immunol..

[71] Murdaca G., Tonacci A., Negrini S., Greco M., Borro M., Puppo F., Gangemi S. (2019). Emerging role of vitamin D in autoimmune diseases: An update on evidence and therapeutic implications. Autoimmun. Rev..

[72] Statello L., Guo C.J., Chen L.L., Huarte M. (2021). Gene regulation by long non-coding RNAs and its biological functions. Nat. Rev. Mol. Cell Biol..

[73] Barnes C.E., English D.M., Cowley S.M. (2019). Acetylation & Co: An expanding repertoire of histone acylations regulates chromatin and transcription. Essays Biochem..

[74] Rafehi H., Balcerczyk A., Lunke S., Kaspi A., Ziemann M., Kn H., Okabe J., Khurana I., Ooi J., Khan A.W. (2014). Vascular histone deacetylation by pharmacological HDAC inhibition. Genome Res..

[75] Ambrosini S., Mohammed S.A., Lüscher T.F., Costantino S., Paneni F. (2020). New mechanisms of vascular dysfunction in cardiometabolic patients: Focus on epigenetics. High Blood Press. Cardiovasc. Prev..

[76] Zarzour A., Kim H.W., Weintraub N.L. (2019). Epigenetic regulation of vascular diseases. Arterioscler. Thromb. Vasc. Biol..

[77] Wagner C.L., Hollis B.W. (2022). The extraordinary metabolism of vitamin D. eLife.

[78] Al-Garawi A., Carey V.J., Chhabra D., Mirzakhani H., Morrow J., Lasky-Su J., Qiu W., Laranjo N., Litonjua A.A., Weiss S.T. (2016). The role of vitamin D in the transcriptional program of human pregnancy. PLoS ONE.

[79] Mulligan M.L., Felton S.K., Riek A.E., Bernal-Mizrachi C. (2010). Implications of vitamin D deficiency in pregnancy and lactation. Am. J. Obstet. Gynecol..

[80] Meems L.M., Mahmud H., Buikema H., Tost J., Michel S., Takens J., Verkaik-Schakel R.N., Vreeswijk-Baudoin I., Mateo-Leach I.V., van der Harst P. (2016). Parental vitamin D deficiency during pregnancy is associated with increased blood pressure in offspring via Panx1 hypermethylation. Am. J. Physiol. Heart Circ. Physiol..

[81] Zhang H., Chu X., Huang Y., Li G., Wang Y., Li Y., Sun C. (2014). Maternal vitamin D deficiency during pregnancy results in insulin resistance in rat offspring, which is associated with inflammation and Iκbα methylation. Diabetologia.

[82] Pike J.W., Meyer M.B. (2012). The vitamin D receptor: New paradigms for the regulation of gene expression by 1,25-dihydroxyvitamin D3. Rheum. Dis. Clin. N. Am..

[83] Izzo M., Carrizzo A., Izzo C., Cappello E., Cecere D., Ciccarelli M., Iannece P., Damato A., Vecchione C., Pompeo F. (2021). Vitamin D: Not just bone metabolism but a key player in cardiovascular diseases. Life.

[84] Apprato G., Fiz C., Fusano I., Bergandi L., Silvagno F. (2020). Natural epigenetic modulators of vitamin D receptor. Appl. Sci..

[85] Saccone D., Asani F., Bornman L. (2015). Regulation of the vitamin D receptor gene by environment, genetics and epigenetics. Gene.

[86] Ionova Z.I., Sergeeva E.G., Berkovich O.A. (2021). Genetic and epigenetic factors regulating the expression and function of the vitamin D receptor in patients with coronary artery disease. Russ. J. Cardiol..

[87] Chen Y., Liu W., Sun T., Huang Y., Wang Y., Deb D.K., Yoon D., Kong J., Thadhani R., Li Y.C. (2013). 1,25-Dihydroxyvitamin D promotes negative feedback regulation of TLR signaling via targeting microRNA-155-SOCS1 in macrophages. J. Immunol..

[88] Bozic M., Álvarez Á., de Pablo C., Sanchez-Niño M.D., Ortiz A., Dolcet X., Encinas M., Fernandez E., Valdivielso J.M. (2015). Impaired vitamin D signaling in endothelial cell leads to an enhanced leukocyte-endothelium interplay: Implications for atherosclerosis development. PLoS ONE.

[89] Al-Rasheed N.M., Al-Rasheed N.M., Bassiouni Y.A., Hasan I.H., Al-Amin M.A., Al-Ajmi H.N., Mohamad R.A. (2015). Vitamin D attenuates pro-inflammatory TNF-α cytokine expression by inhibiting NF-ĸB/p65 signaling in hypertrophied rat hearts. J. Physiol. Biochem..

[90] Gynther P., Toropainen S., Matilainen J.M., Seuter S., Carlberg C., Väisänen S. (2011). Mechanism of 1α,25-dihydroxyvitamin D(3)-dependent repression of interleukin-12B. Biochim. Biophys. Acta.

[91] Joshi S., Pantalena L.C., Liu X.K., Gaffen S.L., Liu H., Rohowsky-Kochan C., Ichiyama K., Yoshimura A., Steinman L., Christakos S. (2011). 1,25-dihydroxyvitamin D(3) ameliorates Th17 autoimmunity via transcriptional modulation of interleukin-17A. Mol. Cell. Biol..

[92] Devaraj S., Yun J.M., Duncan-Staley C.R., Jialal I. (2011). Low vitamin D levels correlate with the proinflammatory state in type 1 diabetic subjects with and without microvascular complications. Am. J. Clin. Pathol..

[93] Zhang Y., Leung D.Y., Richers B.N., Liu Y., Remigio L.K., Riches D.W., Goleva E. (2012). Vitamin D inhibits monocyte/macrophage proinflammatory cytokine production by targeting MAPK phosphatase-1. J. Immunol..

[94] Wang Q., He Y., Shen Y., Zhang Q., Chen D., Zuo C., Qin J., Wang H., Wang J., Yu Y. (2014). Vitamin D inhibits COX-2 expression and inflammatory response by targeting thioesterase superfamily member 4. J. Biol. Chem..

[95] Wang T.J., Pencina M.J., Booth S.L., Jacques P.F., Ingelsson E., Lanier K., Benjamin E.J., D’Agostino R.B., Wolf M., Vasan R.S. (2008). Vitamin D deficiency and risk of cardiovascular disease. Circulation.

[96] Norman P.E., Powell J.T. (2014). Vitamin D and cardiovascular disease. Circ. Res..

[97] Wimalawansa S.J. (2019). Vitamin D deficiency: Effects on oxidative stress, epigenetics, gene regulation, and aging. Biology.

[98] Zhu H., Wang X., Shi H., Su S., Harshfield G.A., Gutin B., Snieder H., Dong Y. (2013). A genome-wide methylation study of severe vitamin D deficiency in African American adolescents. J. Pediatr..

